# Bivalirudin vs. Heparin on Radial Artery Thrombosis during Transradial Coronary Intervention: An Optical Coherence Tomography Study

**DOI:** 10.1155/2020/7905021

**Published:** 2020-09-26

**Authors:** Zijing Liu, Guozhong Wang, Dan Niu, Yongxia Wu, Zixuan Li, Libin Zhang, Guohua Zhu, Qi Hua, Jincheng Guo

**Affiliations:** ^1^Department of Cardiology, Xuanwu Hospital, Capital Medical University, Beijing 10000, China; ^2^Department of Cardiology, Beijing Luhe Hospital, Capital Medical University, Beijing 100000, China

## Abstract

**Objectives:**

This study aimed to evaluate the antithrombotic efficacy between bivalirudin and unfractionated heparin (UFH) on radial artery thrombosis (RAT) during transradial coronary intervention (TRI) by optical coherence tomography (OCT).

**Methods and Results:**

We consecutively reviewed a total of 307 patients who underwent radial artery OCT inspection after TRI in our centre from October 2017 to January 2019; afterwards, 211 screened patients were divided into the UFH group (*n* = 144) and the bivalirudin group (*n* = 67) according to their anticoagulation strategy during TRI. The thrombosis in the radial artery was observed in 51 cases (24.17%) with a median thrombus volume of 0.054 mm^3^ (0.024, 0.334) and median thrombus score of 7 (4, 15). Thrombus occurred in 28 cases in the bivalirudin group with an incidence of 41.8%, which was significantly higher than that in the UFH group (*n* = 23, 16.0%, *P* < 0.001). This difference was even more remarkable after propensity score matching (bivalirudin group *n* = 22, 42.3% vs. UHF group *n* = 11, 13.9%, *P* < 0.001). Multivariate logistic analysis revealed that bivalirudin increased the RAT risk by 3.872 times (95% CI 2.006–8.354, *P* < 0.001) after adjustment for the other predictors.

**Conclusion:**

In this present study, the use of bivalirudin was associated with a higher risk of RAT than UFH. It highlighted UFH should be a more considerable choice to prevent radial artery access thrombosis in TRI.

## 1. Introduction

Radial artery thrombosis (RAT) is a complication of access in transradial coronary intervention (TRI) accompanied by artery trauma or device insertion and is relevant to postprocedural ischemic events in the upper limb [1]. Currently, no precise profile of real-time RAT during TRI has been reported because of observational limitations. Optical coherence tomography (OCT) has proven to be a reliable method to assess intravascular device-related thrombi in coronary intervention in the last decades [2]. A few OCT studies have revealed a remarkably high incidence of RAT from 20.5 to 23.5% during TRI procedures [3, 4]. Although guidelines recommend routine anticoagulant therapy with either unfractionated heparin (UFH) or bivalirudin as an alternative to minimize the risk of thrombosis and ischemic events throughout percutaneous coronary intervention (PCI) [5–7], the efficacy of bivalirudin vs. UFH is still uncertain and controversial in some specific clinical settings including early stent thrombosis in acute coronary syndromes (ACS) [8, 9], as well as catheter thrombogenesis in patients undergoing brachytherapy [10]. Additionally, there have been no attempts to assess the efficacy of these anticoagulants on radial access thrombosis during coronary catheterization. Therefore, the purpose of this study is to evaluate the antithrombotic efficacy of bivalirudin vs. UFH on RAT during TRI by OCT.

## 2. Materials and Methods

### 2.1. Study Population

**Figure 1:**
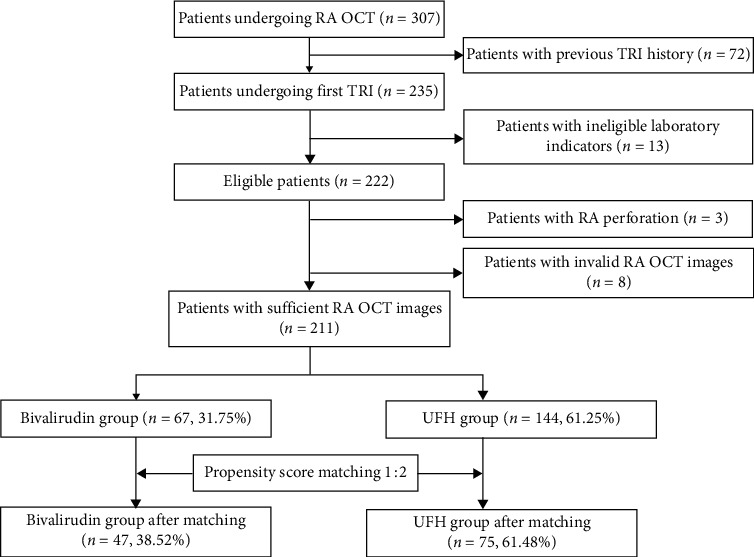
Patient chart of enrollment and propensity score matching. RA: radial artery, OCT: optical coherence tomography, TRI: transradial coronary intervention, and UFH: unfractionated heparin.

We consecutively reviewed a total of 307 patients who underwent radial artery (RA) OCT inspection after TRI and coronary OCT examination for ACS in our centre from October 2017 to January 2019. The exclusion criteria included (1) patients with previous TRI history; (2) hemoglobin < 100 g/L or platelet count < 100 × 10^9^/L; (3) kidney or liver dysfunction with creatinine > 150 *μ*mol/L and/or aminotransferase level > 3 × the upper limit; (4) cases with destructive RA perforation under OCT; and (5) cases without valid OCT images because of serious RA spasm or artifacts. Finally, 211 screened cases with a sufficient number of OCT images were analyzed. These were divided into the UFH group (*n* = 144) and the bivalirudin group (*n* = 67) according to their periprocedure anticoagulation strategy (Figure 1). The patients' clinical characteristics are summarized in Table 1. All patients provided written informed consent before the procedure, and the study protocol was approved by the local ethics committee. This study complied with the Declaration of Helsinki.

### 2.2. Antithrombotic Strategies and Transradial Coronary Intervention

Before TRI, all participants were given 300 mg aspirin and either a loading dose of 180 mg ticagrelor or 300–600 mg clopidogrel orally. Then, according to the options of both operators and patients, the participants were given one of the anticoagulation strategies as follows: (1) intravenous bivalirudin (Salubris Pharmaceuticals Co.) was administered as a bolus dose of 0.75 mg/kg at the beginning of coronary angiography followed by consistent infusion of 1.75 mg/kg/h during the procedure; (2) intravenous administration of a bolus dose of 70–100 IU/kg UFH at the beginning of the procedure, and additional dose was given to maintain the therapeutic activated clotting times 250–300 s (with no planned GP IIb/IIIa receptor antagonist) or 200–250 s (with GP IIb/IIIa receptor antagonist use) according to recommendations [6, 11]. Afterwards, coronary intervention was performed by two experienced operators according to standard protocols, during which the decision for further treatment with injection of tirofiban by a bolous dosage of 10 *μ*g/kg plus a 0.15 *μ*g/kg/min consistent infusion was at the operators' discretion.

### 2.3. Radial Artery OCT Procedure

**Figure 2:**
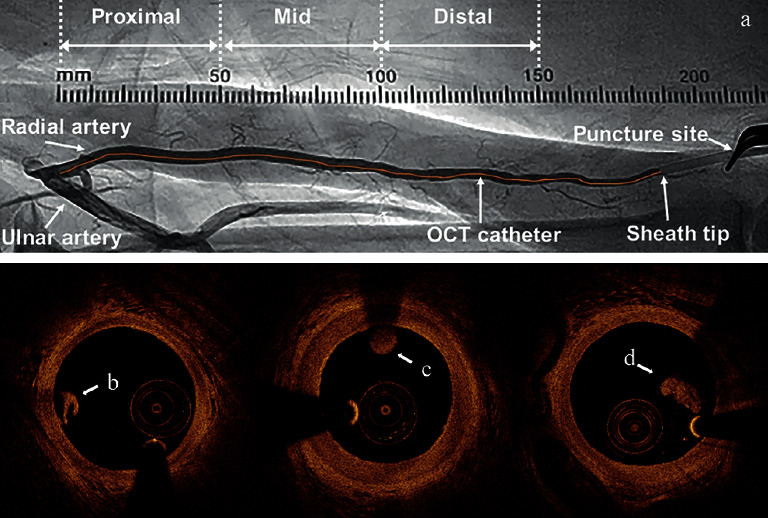
Radial artery OCT imaging method and thrombus under OCT: a, radial artery OCT imaging at proximal, middle, and distal portions, b, white thrombus in the radial artery under OCT, c, red thrombus, and d, mixed thrombus.

Following TRI, the radial sheath (6 French, 16 cm length, Terumo Co., Tokyo, Japan) was immediately withdrawn, leaving 2 cm inside the access site. An antispasmic cocktail of nitroglycerin (0.2 mg) plus verapamil (2.5 mg) was infused intra-arterially before RA OCT imaging acquisition by the C7XR FD-OCT system (St. Jude Medical Inc., St. Paul, MN, USA). Three OCT pullbacks recorded as the proximal, middle, and distal portions of the RA were performed to cover a total length of 15.0 cm of each RA, as shown in Figure 2. Immediately after the completion of TRI, the sheath was removed, and a device compression was applied to achieve a patent hemostasis (proved by reverse Barbeau's test). The compression duration was 4 hours after sheath removal or above if needed.

### 2.4. OCT Image Analysis and Thrombus Measurement

OCT images were analyzed using offline image processing software (permitted by LightLab Imaging, Inc.). Thrombus was defined as a mass attached to the luminal surface or floating within the lumen, based on established criteria. Three patterns of thrombus were identified: red thrombus has high backscattering and optical attenuation [12]; white thrombus has less backscattering and low attenuation and is homogeneous; and mixed thrombus presents with both features, as shown in Figure 2. Quantitative measurement was achieved by thrombus volume (TV) according to previous reports [13, 14]. The thrombus area (TA) was detected by multiple-point trace at each cross section with an interval of 0.2 mm between frames. TV was calculated as TV (mm^3^) = mean TA (mm^2^) × thrombus length (mm). Another semiquantitative evaluation was implemented using the OCT thrombus score (TS), which in brief was the sum of the quadrants containing thrombus in all cross sections [15]. Thrombus burden (TB) was calculated as TB (%) = mean TA (mm^2^)/mean vascular area (mm^2^) × 100%. Both quantitative and qualitative analyses were performed by two OCT readers, and ambiguous images were further examined by a highly experienced analyst. Intraclass correlation coefficient (ICC) was calculated to assess reproducibility between independent observers in terms of TA.

### 2.5. Statistical Analysis

Continuous data were expressed as mean ± SD or median (interquartile range), and comparisons between two groups used *t*-test or Wilcoxon rank-sum test. Categorical variables were presented as counts with percentages and were compared by *χ*^2^ or Fisher's exact test. In order to reduce the potential imbalance in baseline covariates, propensity score matching (PSM) was carried out using a greedy matching protocol with a fixed caliper width of 0.05; then, the bivalirudin and UFH groups were 1 : 2 matched on the basis of the propensity score without replacement. Afterwards, acceptable balance could be indicated by standardized differences (Table 1). Multiple logistic regressions were performed to identify the predictors of RAT, including baseline characteristics, antithrombotic medication, type of coronary intervention (diagnostic coronary angiography and PCI), procedure time, and number of catheters together with acute intima injuries under OCT (including intima tear and intimomedial dissection). A *P* value <0.05 was considered statistically significant. All analyses were performed using SPSS version 23.0 (SPSS, Inc., Chicago, Illinois) and R v3.1.3 (R Development Core Team, 2016).

## 3. Results

### 3.1. Population Characteristics and Propensity Score Matching

A total of 211 participants were enrolled into the analysis, with 144 (68.25%) in the UFH group and 67 (31.75%) in the bivalirudin group. Clinical characteristics such as age, hypertension, operation (diagnostic coronary angiography and/or PCI), and intracoronary use of tirofiban were significantly different between the two groups at baseline. After matching, 122 patients were divided 1 : 2 into the bivalirudin group (*n* = 47) and the UFH group (*n* = 75) based on similar propensity scores; therefore, two comparable cohorts were obtained. Additional procedural characteristics are summarized in Table 1.

### 3.2. Thrombus in the Radial Artery

In the present study, the thrombi in the RA were observed in 51 cases (24.17%) with a median TV of 0.054 mm^3^ (0.024, 0.334), and a median TS of 7 (4, 15). The total calculated thrombus burden was 0.995% (0.556%, 2.039%). More than half were white thrombus (*n* = 29, 56.86%) and distributed in proximal segments (*n* = 32, 62.75%). The details of thrombus types and distributions are illustrated in Table 2.

**Figure 3:**
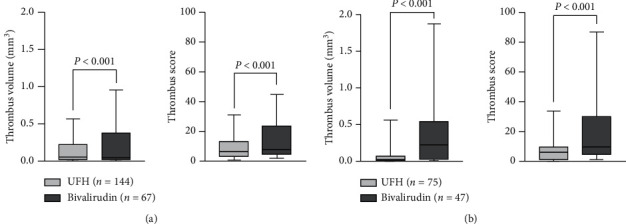
Box and whiskers of thrombus volume and score between the bivalirudin group and the UFH group before and after propensity score matching. The whisker bars represent the 10–90 percentile of total thrombus in each group. (a) Before propensity score matching. (b) After propensity score matching.

Thrombus occurred in 28 cases in the bivalirudin group with an incidence of 41.8% and was significantly higher than that in the UFH group. This difference was even more remarkable after PSM. In the bivalirudin group, 65.71% thrombi (*n* = 23) were found in the proximal portion; however, the distribution difference in the UHF group was not significant. After matching, the distribution difference in the bivalirudin group remained significant. The component of the thrombus was similar between two groups, and white thrombus was in the majority. Total TV in the bivalirudin group was 13.452 mm^3^, and TS was 551, which were both noticeably higher than those in the UFH group, respectively (*P* < 0.001). The trend was also significant after PSM (Figure 3). Detailed quantitative information for thrombus in each portion of the two groups is shown in Table 2.

We performed multivariate logistic regression analysis to identify factors that predicted RAT. In the prematched cohort, anticoagulant, procedure time, and intima injury were predictors for RAT, and bivalirudin increased the RAT risk by 3.872 times (95% CI 2.006–8.354, *P* < 0.001) after adjustment of the other predictors. In the matched cohort, the thrombosis risk of using bivalirudin was still pronounced with an odds ratio of 3.782 (95% CI 1.546–9.253, *P*=0.004), and other predictors were procedure time and intima injury together with the sheath/radial artery diameter ratio (Table 3). The intraclass correlation coefficient (ICC) between two observers was 0.941 (*P* < 0.001).

Nine patients (4.3%) complicated with RAO at 24 hours after TRI in this study, confirmed by ultrasound and/or reverse Barbeau's test. No significance in the incidence of early RAO was observed between the UFH group and the bivalirudin group, 2.8% (4/144) vs. 7.7% (5/67), *P*=0.143. Moreover, none of these patients complained ischemic symptoms of the upper limb in hospitalization.

## 4. Discussion

The main findings of the present study were as follows: compared to UFH, the use of bivalirudin was associated with a higher risk of RAT in transradial coronary catheterization, with white thrombus being predominant and distributed mostly in the proximal segment of the RA, under detection by OCT. Multivariate analysis revealed that using bivalirudin was an independent predictor for RAT, together with acute intima injury and procedure time.

RAT is a complication during transradial intervention and is the main cause for postprocedure RA occlusion. Current reports have demonstrated an incidence from 3 to 10.5% [16, 17] when detected by ultrasound for RA patency after TRI. However, intravascular data for RAT were provided by two OCT studies on RA injury during PCI, suggesting a notably higher frequency of 20.5% and 23.5% [3, 4], and both used UFH for anticoagulation. It is reasonable to infer that the disparity of incidence is due to observational timing as well as the resolution capacity between observational tools. In our study, we found that the general incidence of thrombus was 24.17% (95% CI 18.3–30.0%). In previous studies, no effort was made to determine the thrombus burden of RAT during PCI, which in the present study was low (0.995%) and reflected that the thrombi were mostly microthrombi with a median TV of 0.054 mm^3^ (0.024, 0.334) and a median TS of 7 (4, 15).

Without a clear profile on the mechanism, all the factors in Virchow's triad including endothelial injury, blood flow stasis, and hypercoagulability could share responsibility in RAT. For example, (1) local arterial endothelium trauma caused by device manipulation in RA access was an independent predictor for RAT in our multivariate regression; (2) as a hydrodynamic result, stasis of blood flow concomitant to sheath insertion was inevitable, and sheath diameter/radial diameter (S/R) ratio may be one of the influencing factors [18]; (3) contact activation of coagulation could be triggered by the thrombogenic device as a foreign body and worsened by the hypercoagulability in ACS patients [19]. As a consequence, thrombus formation can be observed as early as 15 mins immediately after the introduction of the catheter in the RA [20].

In the present study, the superiority of antithrombotic efficacy demonstrated by UFH over bivalirudin in the RA may be due to multiple complicated reasons. First, a comparison between the pharmacodynamic characteristics of the research medicine may be helpful. Bivalirudin binds to circulating thrombin in a high dose- or concentration-dependent manner with a small blood volume and short cleavage half-life [21, 22]. In contrast, UFH acts in a nonspecific and nonlinear dose response manner [9]. In addition, it also has the unique property of inactivating factor XIIa and kallikrein generated by the artificial device surface [19]. Thus, UFH showed more efficiency in situations when local drug delivery is difficult to sustain to achieve an optimal anticoagulation intensity in target regions such as the RA lumen and catheter surfaces. On the contrary, the outer diameter of a normal 6Fr sheath (2.62 mm) in our study was approximately equivalent to the inner diameter of the RA (2.79 ± 0.49 mm); thus, the tight contact between the device and the vessel wall may result in the reduction of blood volume and stasis of blood flow, which may lead to a decrease of bivalirudin dose, made even worse by cleavage of the medicine. In vitro results have proven that the antithrombotic efficacy of bivalirudin on catheter thrombosis depends on its continuous infusion [23], which may be insufficient considering the less fluidity of blood in this situation. As a result, the stoichiometric anticoagulant effect of bivalirudin might be overwhelmed when there is consistent coagulation activity caused by exposure to the artificial surface of the device, as learned from the lessons of acute stent thrombosis and coronary device thrombosis [8, 24]. In addition, the procedure time was an important consideration to RAT based on the result from our multiple regression, and in most cases, UFH concentration may be sustained long enough for the PCI procedure after initial injection, as opposed to bivalirudin because of apparent half-life [9].

However, in the aforementioned study, bivalirudin and heparin presented similar efficacy in preventing RAO at 4–8 weeks after TRI [25]. One of the possible explanations for this discrepancy between the incidence of RAT and RAO is that, in our study, most thrombi detected by OCT were microthrombi with a low thrombus burden, which may be too tiny to block blood or bring about any definite clinical sequelae. As a result, there is a chance that the incidence of RAT during TRI has been underestimated in previous studies. For example, data from ACUITY and SWITCH III trials showed that no device-associated thrombus could be documented under routine angiography [26, 27]. Currently, OCT has shed light on the assessment of intravascular thrombus and evaluating efficacy of antithrombotic drugs in vivo [2], and it is reasonable that growing evidence for intraprocedure thrombosis will draw attention in the future.

To our knowledge, this is the first study to evaluate the anticoagulant effect of bivalirudin vs. UFH on RAT during transradial coronary intervention by OCT. We hope it may provide a new clue for selection of anticoagulation strategies for practice.

### 4.1. Limitations

The first limitation of this study is that it did not have a prospective randomized design. Therefore, propensity score matching was performed to adjust the baseline characteristics between groups to control for potential bias. Second, the lack of long-term follow-up in the present study may lead to underrating of the relationship between RAT and RAO. Thus, long-term follow-up should be taken to determine the outcomes on RAO in cases with observational thrombi under OCT. Third, the activated clotting time was not routinely monitored in this study when we used a recommended weight-based dose of UFH for PCI in accordance with guidelines [6]. This may potentially have the risk of missing optimal anticoagulative dosing of UFH. Finally, one of the maneuver disadvantages that flushing ahead of OCT image wire pullback might have a chance to remove a tiny thrombus to distal and lead to an observation deficiency.

## 5. Conclusions

In this study, the use of bivalirudin in transradial coronary angiography or PCI was associated with a higher risk of RAT compared with UFH detected by OCT. No difference was observed in RAO after the procedure. The results highlighted that UFH may be preferable over bivalirudin to prevent RAT in the local radial artery access.

## Figures and Tables

**Table 1:** Baseline characteristics of the study population.

	Unmatched cohort (*n* = 211)			Matched cohort (*n* = 119)			Std. mean diff.	
	Bivalirudin (*n* = 67)	UFH (*n* = 144)	*P* value	Bivalirudin (*n* = 47)	UFH (*n* = 75)	*P* value	Before	After
Propensity	0.431	0.265		0.376	0.375		0.909	0.002
Age (years)	60.4 ± 12.6	54.9 ± 11.9	0.003	58.9 ± 13.2	57.3 ± 11.0	0.445	0.432	0.040
Male gender	51 (76.1%)	125 (86.8%)	0.052	44 (84.6%)	66 (83.5%)	0.870	0.249	−0.090
Body mass index (kg/m^2^)	25.6 (23.9–27.7)	25.9 (23.6–28.4)	0.996	25.4 (23.5–28.9)	25.7 (23.8–29.0)	0.873	0.010	0.021
Diabetes	22 (32.8%)	40 (27.8%)	0.453	16 (30.8%)	27 (34.2%)	0.684	0.107	−0.061
Hypertension	45 (67.2%)	70 (48.6%)	0.012	33 (63.5%)	49 (62.0%)	0.868	0.392	−0.081
Hyperlipidemia	55 (82.1%)	103 (71.5%)	0.100	40 (76.9%)	60 (75.9%)	0.898	0.273	−0.050
Current smoking	39 (58.2%)	98 (68.1%)	0.163	34 (65.4%)	52 (65.8%)	0.959	−0.198	0.116
PVD	3 (4.5%)	5 (3.5%)	0.711	3 (5.8%)	4 (5.1%)	1.000	0.048	0.046
Family history	12 (17.9%)	18 (12.5%)	0.295	8 (15.4%)	14 (17.7%)	0.726	0.140	−0.075
Creatinine (*μ*mol/L)	75.00 (64.00–84.00)	78.00 (66.25–89.75)	0.130	75.00 (67.50–86.00)	74.00 (63.00–88.00)	0.510	−0.240	0.090
Diagnoses			0.065			0.702	−0.433	−0.112
MI	65 (97.0%)	129 (89.6%)		50 (96.2%)	74 (93.7%)			
UAP	2 (3.0%)	15 (10.4%)		2 (3.8%)	5 (6.3%)			
Multivessel disease, *N* (%)	86.6%	78.5%		82.7%	82.3%			
Antiplatelet medicine								
Aspirin	67 (100%)	144 (100%)		47 (100%)	75 (100%)			
Clopidogrel	31 (46.3%)	58 (40.3%)		23 (44.2%)	30 (38.0%)			
Ticagrelor	36 (53.7%)	86 (59.7%)		29 (55.8%)	49 (62.0%)			
Tirofiban	24 (35.8%)	31 (21.5%)	0.028	17 (32.7%)	16 (20.3%)	0.109	0.296	0.159
Total dose of UFH (IU)	8000 (7025, 9200)			8000 (7200, 9000)				
Procedure type			0.023			1.000	0.730	0.079
CAG	1 (1.5%)	15 (10.4%)		1 (1.9%)	3 (3.8%)			
PCI	66 (98.5%)	129 (89.6%)		51 (98.1%)	76 (96.2%)			
Procedure time (min)	73.00 (61.00–87.00)	72.00 (57.00–88.75)	0.557	72.00 (61.25–81.50)	69.00 (56.00–84.00)	0.193	0.008	0.139
Number of total catheters	2.07 ± 0.745	1.94 ± 0.936	0.132	1.97 ± 0.698	2.02 ± 1.019	0.899		
Number of diagnostic catheters	0.81 ± 0.625	0.79 ± 0.616	0.907	0.76 ± 0.582	0.83 ± 0.648	0.552		
Number of guiding catheters	1.26 ± 0.469	1.15 ± 0.557	0.022	1.22 ± 0.414	1.19 ± 0.627	0.271		

PVD: peripheral vascular disease, MI: myocardial infarction, UAP: unstable angina pectoris, CAG: coronary angiography, and PCI: percutaneous coronary intervention.

**Table 2:** Thrombus in the RA during coronary intervention.

		Before PSM			After PSM		
		Bivalirudin (*n* = 67)	UFH (*n* = 144)	*P* value	Bivalirudin (*n* = 47)	UFH (*n* = 75)	*P* value
Proximal	Thrombus	23 (34.3%)	9 (6.3%)		18 (34.6%)	2 (2.5%)	<0.001
	Type (white/red/mix)	11/9/3	5/1/3		8/8/2	1/1/0	
	Total TV (mm^3^)	12.177	2.517	<0.001	12.007	0.085	<0.001
	Total TS	444	99		421	9	<0.001
	TB (%)	1.36 (0.65, 2.54)	0.99 (0.68, 2.44)		1.37 (0.80, 3.55)	0.81 (0.67, ‐)	<0.001

Middle	Thrombus	8 (11.9%)	10 (6.9%)	0.227	7 (13.5%)	5 (6.3%)	0.218
	Type (white/red/mix)	4/2/2	6/2/2		3/2/2	2/2/1	
	Total TV (mm^3^)	0.892	1.511	0.223	0.864	1.171	0.173
	Total TS	68	98	0.224	60	69	0.179
	TB (%)	0.82 (0.49, 1.46)	0.73 (0.36, 1.24)	0.219	0.96 (0.62, 1.53)	0.98 (0.37, 2.18)	0.167

Distal	Thrombus	4 (6.0%)	7 (4.9%)	0.746	4 (7.7%)	5 (6.3%)	0.740
	Type (white/red/mix)	2/1/1	4/2/1		2/1/1	3/2/0	
	Total TV (mm^3^)	0.384	0.192	0.690	0.384	0.076	0.684
	Total TS	39	28	0.683	39	19	0.699
	TB (%)	0.80 (0.43, 2.59)	0.46 (0.33, 1.19)	0.722	0.80 (0.43, 2.59)	0.41 (2.92, 0.83)	0.723

Total	Thrombus	28 (41.8%)	23 (16.0%)	<0.001	22 (42.3%)	11 (13.9%)	<0.001
	Type (white/red/mix)	15/7/6	14/4/5		11/6/5	6/4/1	
	Total TV (mm^3^)	13.452	4.219	<0.001	13.254	1.332	<0.001
	Total TS	551	225	<0.001	520	97	<0.001
	TB (%)	1.32 (0.59, 2.63)	0.88 (0.39, 1.66)	<0.001	1.58 (0.67, 3.95)	0.72 (0.37, 1.22)	<0.001

TV: thrombus volume, TS: thrombus score, and TB: thrombus burden.

**Table 3:** Independent predictors of radial artery thrombosis by multivariate logistic regression.

	Before PSM			After PSM		
	OR	95% CI	*P* value	OR	95% CI	*P* value
Intima injury	3.955	1.937–8.075	<0.001	2.759	1.132–6.725	0.026
Anticoagulant	3.872	1.886–7.950	<0.001	3.782	1.546–9.253	0.004
Procedure time	1.021	1.008–1.034	0.003	1.026	1.003–1.049	0.026
*S*/*R* ratio	1.827	0.863–3.866	0.115	2.947	1.122–7.741	0.028

PSM: propensity score matching, OR: odds ratio, and *S*/*R* ratio: sheath diameter/radial artery diameter ratio.

## Data Availability

The datasets used or analyzed during the current study are available from the corresponding author on reasonable request.
